# Supplementary material for: Predation and fragmentation portrayed in the statistical structure of prey time series
Source: BMC Ecol. 2009 May 6;9:10. doi: 10.1186/1472-6785-9-10 (PMC2689204; doi:10.1186/1472-6785-9-10)
Supplement: Additional file 2 — Voles and related classes ODDox Documentation. ODDox documentation of the agent-based model (ALMaSS) applied by Hendrichsen et al. The documentation is started by activating main.html. [file 1472-6785-9-10-S2.zip › Vole_ODDox/globals.html]

ALMaSS ODDox: Class Members

- Main Page
- Related Pages
- Classes
- Files

- File List
- File Members

- All
- Functions
- Variables
- Typedefs
- Enumerations
- Enumerator
- Defines

- \_
- a
- b
- c
- d
- f
- g
- h
- i
- l
- m
- n
- o
- p
- r
- s
- t
- u
- v
- w

Here is a list of all file members with links to the files they belong to:

### - \_ -

- \_\_CIPEGRIDSIZE
  : VolePopulationManager.cpp- \_\_GENDEBUG
    : GeneticMaterial.cpp- \_\_lgridsize
      : PopulationManager.h- \_\_SpecificPesticideEffectsVinclozolinLike\_\_
        : VolePopulationManager.cpp
        , Vole\_all.cpp
        , vole\_all.h- \_CRT\_SECURE\_NO\_DEPRECATE
          : VolePopulationManager.cpp
          , farm.cpp
          , GeneticMaterial.cpp
          , PopulationManager.cpp
          , croprotation.cpp
          , Predators.cpp
          , Vole\_all.cpp- \_CRTDBG\_MAP\_ALLOC
            : farm.cpp
            , PopulationManager.cpp

---

Generated on Thu Jan 22 14:13:48 2009 for ALMaSS ODDox by 
 1.5.6
